# Dupilumab-Related Hypereosinophilia in Patients Treated for Type 2 Diseases: Evidence from a 24-Month Prospective Real-Life Study

**DOI:** 10.3390/jcm15041525

**Published:** 2026-02-14

**Authors:** Ilaria Mormile, Daniele La Prova, Paolo Pezzella, Giuliano Di Caprio, Amato de Paulis, Elena Cantone, Aikaterini Detoraki

**Affiliations:** 1 Department of Translational Medical Sciences, Federico II University, 80131 Naples, Italy; depaulis@unina.it; 2 Post-Graduate Program in Clinical Immunology and Allergy, University of Naples Federico II, 80131 Naples, Italy; danielelaprovadlp@gmail.com (D.L.P.); dicapriogiuliano@gmail.com (G.D.C.); 3 Department of Neuroscience, Reproductive and Odontostomatological Sciences-ENT Section, University of Naples Federico II, 80131 Naples, Italy; ppezzella22@gmail.com; 4 Center for Basic and Clinical Immunology Research (CISI), University of Naples Federico II, 80131 Naples, Italy; 5 WAO Center of Excellence, 80131 Naples, Italy; 6 Division of Internal Medicine and Clinical Immunology, Department of Internal Medicine and Clinical Complexity, University of Naples Federico II, 80131 Naples, Italy; kate.detoraki@gmail.com; 7 Department of Pharmacy and Health and Nutrition Sciences, University of Calabria, 87036 Rende, Italy; cantoneent@gmail.com; 8 Otolaryngology Unit, Annunziata Hospital, 87100 Cosenza, Italy

**Keywords:** asthma, chronic rhinosinusitis with nasal polyps, eosinophilia, eosinophils, dupilumab, hypereosinophilia

## Abstract

**Background/Objectives:** Dupilumab is a human monoclonal antibody that targets both IL-4 and IL-13 signaling. Eosinophilia has been reported as a potential adverse event in treated patients in randomized controlled trials and 12-month real-life studies. This real-life, 24-month prospective study investigated the prevalence of eosinophilia, its consequences, and the effectiveness of dupilumab in a cohort of patients with severe asthma, chronic rhinosinusitis with nasal polyps, and atopic dermatitis. **Methods:** A total of 66 adult patients treated with dupilumab were included in this study. ACT, SNOT-22, and Smell-VAS, EASI, and absolute blood eosinophil count (AEC) were assessed according to the type of diagnosis at baseline (T0), after 6 (T6), 12 (T12), 18 (T18), and 24 (T24) months post-dupilumab initiation. **Results:** All patients experienced significant improvement in both symptoms and disease control following dupilumab treatment. A total of 27 out of 66 (40.9%) patients developed eosinophilia within six months of treatment (AEC T6 mean ± SD 0.67 ± 0.68 10^9^/L). Eosinophilia was generally mild and lasted on average for six months (AEC T12 mean ± SD 0.66 ± 0.65 10^9^/L) with AEC normalization after 18 months of treatment (AEC T18 mean ± SD 0.58 ± 0.48 10^9^/L). Ten patients (15.15%) developed hypereosinophilia, but no symptoms or signs of eosinophilic-related organ damage have been observed, with no need for dupilumab discontinuation. **Conclusions:** Dupilumab-related eosinophilia was common, generally mild, and transient, whereas persistent hypereosinophilia occurred in a small group of patients in the absence of symptoms or signs of eosinophilic damage.

## 1. Introduction

Dupilumab is a human monoclonal antibody targeting both IL-4 and IL-13 signaling approved for the treatment of type 2 severe asthma, chronic rhinosinusitis with nasal polyps (CRSwNP), atopic dermatitis, and eosinophilic esophagitis. Adverse reactions to dupilumab include injection site reactions, conjunctivitis, arthralgia, and oral herpes [1]. An increased absolute blood eosinophil count (AEC) ≥ 0.5 × 10^9^/L has also been commonly reported [2,3,4,5,6,7]. Conversely, dupilumab-induced hypereosinophilia, defined as AEC ≥ 1.5 × 10^9^/L, is reported as rare, early, and transient [1,5]. However, the percentages of occurrence described in clinical trials and real-life studies vary widely (from 4% to 55%) [5,8,9,10]. Higher percentages have been generally reported in real-life studies compared to clinical trials [8,9,10,11,12]. Moreover, to the best of our knowledge, no studies on dupilumab-induced eosinophilia lasting more than 12 months are available. Blood eosinophilia observed during dupilumab therapy is presumably due to the blockade of the IL-4/IL-13 pathway with subsequent inhibition of cell migration into tissues [4]. Chemokines and adhesion molecules regulate adhesion of circulating eosinophils to blood vessels with receptors on the endothelium, like VCAM-1, which are regulated by IL-4 [4,13]. Once eosinophil have entered tissues, their migration is guided by chemokines such as eotaxin, IL-5, and IL-13 [4,14]. In addition, IL-4 and IL-13 regulate the expression of eosinophil chemotactic factors (e.g., eotaxin-1, eotaxin-3, MCP-4) [15,16]. Thus, dupilumab, by blocking IL-4 and IL-13, may inhibit eosinophil migration and trafficking, and transient blood eosinophil counts may be observed [4]. Another hypothesis is that oral corticosteroid reduction or discontinuation in patients under dupilumab may contribute to eosinophilia [17]. Eosinophil-related organ damage is not easily predicted from eosinophil counts, as organ or tissue damage is not always present. However, despite being generally considered benign, cases of eosinophilic pneumonia and eosinophilic granulomatosis with polyangiitis (EGPA) reported during dupilumab treatment may imply that increased AEC may not be limited to the periphery [18,19,20]. According to recent studies, a cutoff of ≥1.5 × 10^9^/L eosinophils is considered a critical value for requiring a further diagnostic workup in a specialist setting to exclude eosinophilic disorders (hypereosinophilic syndrome (HES), EGPA, eosinophilic pneumonia) [21,22,23]. Specific recommendations for the management of dupilumab-associated hypereosinophilia are usually adopted empirically, based on a literature review and an algorithmic approach to clinical management [5]. The aim of our real-life prospective observational study is to assess baseline characteristics, prevalence, timing, response to treatment, and practical management in a cohort of patients presenting eosinophilia during dupilumab therapy over a 24-month follow-up period.

## 2. Results

### 2.1. Demographics and Baseline Clinical Features

A total of 66 adult patients (33 males (50%) and 33 females (50%)) treated with dupilumab were included in this study (Table 1).

The average age at enrollment was 52.9 ± 17.4 years (range 18–84). The cohort was Caucasian except for one female patient who was Asian. Dupilumab was prescribed in 54 (81.81%) patients for CRSwNP, in 6 (9.09%) patients for severe asthma, and in 6 patients (9.09%) for atopic dermatitis. The presence of type 2 comorbidities at baseline was investigated for each patient. Among patients receiving dupilumab for CRSwNP, 33 (61.11%) also had bronchial asthma, 27 (50%) had allergic rhinitis, 10 (18.51%) had allergic conjunctivitis, 1 (1.85%) had a food allergy, and no patient had atopic dermatitis. Among the six patients receiving dupilumab for severe asthma, 5 (83.33%) also had allergic rhinitis, 2 (1.51%) had a food allergy, and none had CRSwNP, allergic conjunctivitis, or atopic dermatitis. Among the six patients receiving dupilumab for atopic dermatitis, three (50%) also had bronchial asthma, four (66.66%) had allergic rhinitis, and all patients (100%) had a food allergy; no patient had CRSwNP.

### 2.2. Clinical Outcomes Following Dupilumab Treatment

**Figure 1:**
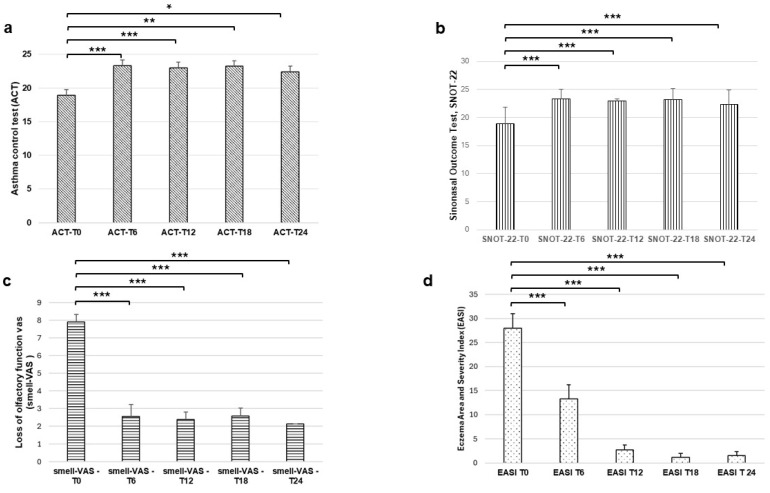
Clinical outcomes in our cohort of patients treated with dupilumab. Asthma Control Test, ACT (**a**), Sinonasal Outcome Test, SNOT-22 (**b**), visual analog scale (VAS) score for the evaluation of loss of olfactory function, Smell-VAS (**c**) and Eczema Area and Severity Index, EASI (**d**), collected at baseline, after 6, 12, 18 and 24 months of dupilumab treatment. * *p* < 0.05; ** *p* < 0.01; *** *p* < 0.001.

ANOVA conducted on ACT revealed a significant difference among T0, T6, T12, T18, and T24 [F(4, 152) = 11.9; *p* < 0.001; Figure 1], demonstrating in all patients a significant improvement in both asthma control and symptoms following dupilumab treatment. Post hoc comparisons revealed a higher ACT score at T6 than at T0 (post hoc *p* < 0.001), suggesting that the improvements in our cohort were significant already after six months of treatment. These results were maintained after 12 months (ACT T0 vs. ACT T12 post hoc *p* < 0.001), 18 months of treatment (ACT T0 vs. ACT T18 post hoc *p* = 0.003) and 24 months (ACT T0 vs. ACT T24 post hoc *p* = 0.017). No significant difference was observed between T6 and T12 (post hoc *p* = 0.996), between T6 and T12 (post hoc *p* = 0.939), and between T12 and T18 (post hoc *p* = 0.465) (Figure 1a).

Dupilumab was also effective in improving nasal symptoms as shown by the significant difference revealed by ANOVA conducted on SNOT-22 [F(4, 164) = 25; *p* < 0.001; Figure 1b] and Smell-VAS [F(4, 112) = 32,9; *p* < 0.001; Figure 1c] among T0, T6, T12, T18, and T24. Post hoc comparisons revealed a higher score at both SNOTT-22 and Smell-VAS for T6 compared to T0 (post hoc *p* < 0.001), suggesting that the improvements in our cohort were significant already after six months of treatment also for nasal symptoms and maintained after 12 months (SNOT-22 T0 vs. SNOT-22 T12 post hoc *p* < 0.001; Smell-VAS T0 vs. Smell-VAS T12 post hoc *p* < 0.001), 18 months of treatment (SNOT-22 T0 vs. SNOT-22 T18 post hoc *p* < 0.001; Smell-VAS T0 vs. Smell-VAS T18 post hoc *p* < 0.001), and 24 months (SNOT-22 T0 vs. SNOTT-22 T24 post hoc *p* < 0.001; Smell-VAS T0 vs. Smell-VAS T24 post hoc *p* < 0.001) (Figure 1b,c).

Finally, ANOVA conducted on Eczema Area and Severity Index (EASI) revealed a significant difference among T0, T6, T12, T18, and T24 [F(4, 20) = 48.2; *p* < 0.001; Figure 1d], demonstrating dupilumab effectiveness on atopic dermatitis in our cohort. Post hoc comparisons revealed a significant decrease in the EASI score already after six months of treatment (post hoc *p* < 0.001), which was maintained after 12 months (post hoc *p* = 0.002), 18 months (post hoc *p* = 0.002), and 24 months (post hoc *p* = 0.001) of treatment (Figure 1d).

### 2.3. Absolute Peripheral Eosinophil Count Assessment

**Figure 2:**
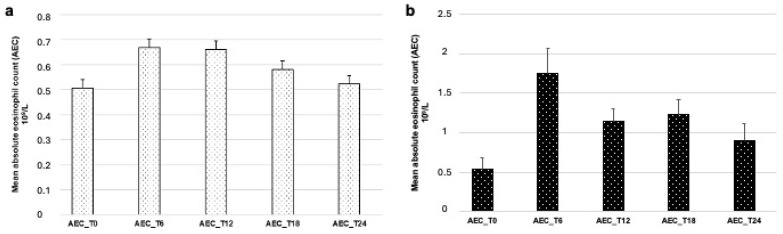
Mean absolute eosinophil count (AEC, 10^9^/L) showing patients developing eosinophilia (**a**) and hypereosinophilia (**b**) at different follow-up visits after dupilumab treatment.

A total of 27 out of 66 (40.9%) patients developed eosinophilia (AEC ≥ 0.5 × 10^9^/L) within six months of dupilumab treatment (AEC T6 mean ± SD 0.67 ± 0.68 10^9^/L). The eosinophilia was generally mild and lasted on average for six months (AEC T12 mean ± SD 0.66 ± 0.65 10^9^/L) with AEC normalization after 18 months of treatment in most patients (AEC T18 mean ± SD 0.58 ± 0.48 10^9^/L). ANOVA conducted on eosinophil levels at T0, T6, T12, T18 and T24 did not reveal a significant difference among the visits [F(4, 172) = 1.16; *p* = 0.328] (Figure 2a).

Ten patients (15.15%) developed hypereosinophilia following dupilumab treatment. In particular, six patients (9.06%) developed hypereosinophilia at T6, two patients (3.03%) at T12, and two (3%) at T18. No new-onset hypereosinophilia was recorded at T24. In four of these patients, hypereosinophilia was reported as persistent, present at all AEC measurements (T6, T12, T18, T24). ANOVA conducted on AEC in patients presenting with hypereosinophilia revealed a significant difference among the AEC determinations [F(4, 32) = 3.28; *p* = 0.023] (Table 2).

However, post hoc comparisons with Tukey correction revealed no statistically significant differences among any of the AEC determinations (Figure 2b).

No symptoms or signs of eosinophilic-related organ damage were observed. None of these patients discontinued dupilumab, as its administration was safe and associated with improvement in clinical outcomes.

**Figure 3:**
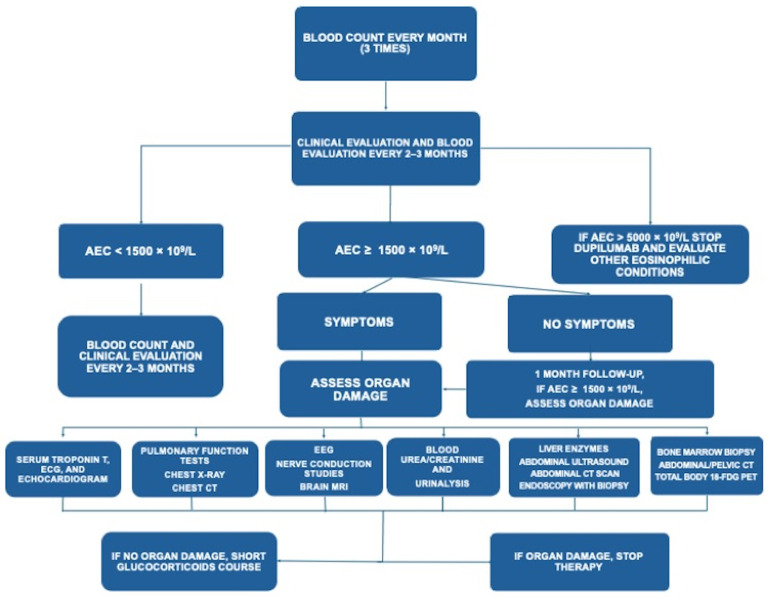
Adopted flow chart for the management of hypereosinophilia in course of dupilumab therapy.

However, in patients who developed hypereosinophilia, stepwise monitoring was performed to exclude eosinophilic disorders or secondary causes, according to the flow chart shown in Figure 3.

### 2.4. Clinical Features of Patients with Dupilumab Induced-Hypereosinophilia

Clinical characteristics of patients who developed hypereosinophilia following dupilumab initiation were evaluated.

In almost all patients developing hypereosinophilia (9 out of 10 patients, 90%), dupilumab was prescribed for CRSwNP. One patient underwent dupilumab treatment for bronchial asthma. No significant statistical difference was found between basal AEC between patients developing AEC ≥ 1.5 × 10^9^/L vs. patients with AEC < 1.5 × 10^9^/L [t(62) = −0.741; *p* = 0.462], suggesting that patients developed hypereosinophilia regardless of their basal eosinophil count.

No significant statistical difference was found [t(57) = 0.269; *p* = 0.789] between basal total IgE in patients developing AEC ≥ 1.5 × 10^9^/L (mean total IgE 288 ± 288) vs. patients with AEC < 1.5 × 10^9^/L (mean total IgE 331 ± 499), suggesting that patients developed hypereosinophilia irrespectively of their basal total IgE levels.

Only 17 out of 66 (25.75%) patients had positive specific IgE and/or skin prick tests (SPTs) for aeroallergens (Table 1). Most prevalent allergens in our cohort were (Gramineae grass pollen (Gramineae mix/Phleum Pratense/Cynodon Dactilon), resulting positive in 10 out of 17 patients (58.8%), and house dust mites (*Dermatophagoides pteronyssinus* and *Dermatophagoides farinae*) in 7 patients (41.17%). An increase in AEC > 0.5 × 10^9^/L was observed in almost all patients sensitized to aeroallergens (15 out of 17 patients, 88.23%) even though an increase to >1.5 × 10^9^/L was shown by only 4 patients.

### 2.5. Adverse Events During Dupilumab Treatment

The adverse reactions to dupilumab were generally mild in our cohort and did not require drug withdrawal. The frequency is summarized in Table 3.

**Figure 4:**
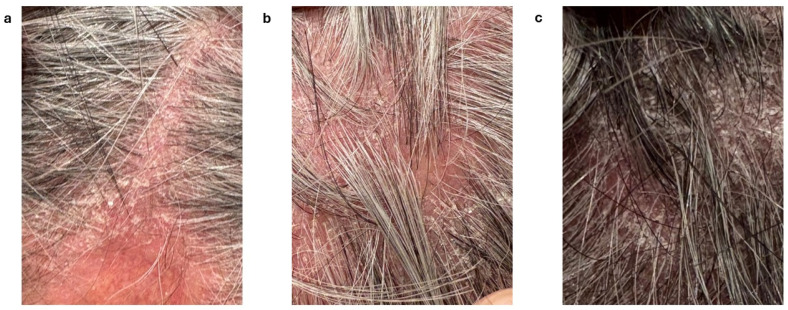
Psoriatic eruption on the patient’s scalp. Well-demarcated erythematous plaque along the scalp part line, covered by thick, adherent whitish–silvery scales with lamellar desquamation; hair density is preserved with no evidence of scarring (**a**). Extensive erythematous scalp plaque with abundant multilayered white scales distributed over the scalp surface and proximal hair shafts; hair shafts are intact, with no signs of cicatricial alopecia (**b**). Scalp area showing persistent psoriatic involvement characterized by erythema and fine whitish scaling, with coexisting darkly pigmented hair shafts interspersed among gray hairs, consistent with reversal of hair graying (**c**).

Notably, one patient undergoing dupilumab treatment for CRSwNP presented with scalp psoriasis (Figure 4a,b) with associated reversal of gray hair (Figure 4c).

The patient presented a complete response to dupilumab in terms of nasal symptoms and outcomes. Of note, his AEC was normal both at the baseline and follow-up visits.

## 3. Discussion

Adverse events during dupilumab treatment are generally mild and well tolerated, including injection site reactions, arthralgia, and, in patients treated for atopic dermatitis, conjunctivitis [24,25]. An increase in AEC is also considered common even if real-life studies evaluating the development of hypereosinophilia (i.e., AEC ≥ 1.5 × 10^9^/L) are currently mainly conducted over a 12-month follow-up period and limited in populations treated with dupilumab for specific indications (e.g., CRSwNP) [10,25]. However, a narrative review by Caminati et al. [5] analyzing the existing literature on this topic reported that hypereosinophilia may occur in patients treated with dupilumab irrespective of the indication for which the drug was prescribed and may spontaneously resolve over time. The dupilumab-induced increase in AEC has been reported as transient, not affecting drug efficacy, and rarely associated with clinical consequences [2]. On the other hand, a small number of case reports described patients with clinical signs and symptoms related to eosinophilic damage of different severity [26,27,28,29]. Indeed, although generally considered benign, cases of progression to overt eosinophil-associated disorders, including hypereosinophilic syndrome and EGPA, have been reported [27,30]. In our cohort, eosinophilia (i.e., AEC ≥ 0.5 × 10^9^/L) occurred on average after 6 months of treatment with dupilumab, presenting generally in a mild, transient form. Indeed, eosinophils returned to the reference range over the following 6 months. In addition, no patient presented symptoms or signs of eosinophilic-related organ damage nor required dupilumab discontinuation.

Kemp et al. [25] followed a cohort of patients treated with dupilumab for CRSwNP for 12 months, reporting that hypereosinophilia developed in a minority of patients (16.2% with AEC ≥ 1.5 × 10^9^/L and 1.7% ≥ 3.0 × 10^9^/L) and was mostly mild and transient. In addition, baseline AEC was significantly higher in patients who developed hypereosinophilia [25]. Similarly, a recent study [10] confirmed that dupilumab-induced blood eosinophilia is mostly transient and harmless. In particular, the authors analyzed the eosinophilia onset temporal pattern, revealing that patients predominantly developed early-onset temporary eosinophilia. Caminati et al. [9] reported that the probability of developing hypereosinophilia was 3.3 times higher in patients with baseline AEC between 0.5 and 1.5 × 10^9^/L. However, in our cohort AEC basal level was not significantly higher in patients with AEC ≥ 1.5 × 10^9^/L than in those with AEC < 1.5 × 10^9^/L. Total IgE at baseline did not show a significant difference between patients who developed hypereosinophilia and those who did not during dupilumab treatment. Similarly, specific sensitization to aeroallergens did not appear to be associated with the development of hypereosinophilia, even though almost all patients sensitized to inhalants developed an increase in AEC > 0.5 × 10^9^/L. These results are a starting point to be corroborated by further analysis in a broader cohort.

The transient increase in eosinophil counts observed following dupilumab therapy is thought to result from IL-4/IL-13–mediated inhibition of eosinophil migration from the bloodstream into tissues [5,31,32,33]. The self-limiting nature of this phenomenon is not fully understood. One possible explanation is that overall suppression of type 2 inflammation leads to reduced production of IL-5, GM-CSF, and other survival signals over time, gradually decreasing bone marrow eosinophil output and shortening eosinophil survival [32,34]. Furthermore, unlike hypereosinophilic syndromes, there is no autonomous clonal or cytokine-driven process sustaining eosinophilia [31,35]. Once the transient redistribution imbalance resolves, eosinophil counts decline spontaneously without the need for intervention.

So far, there are no specific recommendations on the management of dupilumab-associated hypereosinophilia. A practical flow chart (Figure 3) was adopted in this study to manage patients with this complication and investigate potential eosinophil-related morbidities. Indeed, in patients presenting with an increase in AEC during dupilumab administration, it is prudent to adopt close monitoring for signs and symptoms suggestive of eosinophil-related organ involvement. A comparison between baseline eosinophilia, often present in patients with asthma and CRSwNP, and eosinophilia occurring during dupilumab therapy should be performed, taking into account increases of at least 50% in eosinophilic blood count, as variations of 20% may be considered dropping into the physiological variability [36,37]. In cases of severe eosinophilia detected before drug initiation, a detailed diagnostic workup should be performed to identify preexisting eosinophilic disorders and exclude secondary causes of eosinophilia [21,22,23,38,39]. In addition, once eosinophilic diseases other than asthma and CRSwNP are ruled out, the choice of biologic therapy should probably be oriented towards other targeted treatments (e.g., mepolizumab, benralizumab) [40,41,42,43]. In patients eligible for dupilumab treatment, after the baseline evaluation, we suggest determining AEC every month for the first 3 months, then every 3 months thereafter. In case of an increase in AEC ≥ 1.5 × 10^9^/L, AEC is repeated after 2 weeks, and if the trend is towards augmentation, organ assessment is performed, evaluating kidney, liver, pancreatic, and cardiac function/damage, as well as skin and respiratory signs and symptoms [21,22,23,44,45]. If damage is present and a diagnosis of an eosinophilic disorder is reached, dupilumab discontinuation is mandatory, and specific therapy should be prescribed. In case of hypereosinophilia without organ damage, dupilumab therapy can be continued, and in case of persistent eosinophilia, a multidisciplinary evaluation should be performed. In case of asymptomatic patients, chest X-ray, echocardiogram, abdominal ultrasound, neurological evaluation, and determination of MPO-ANCA, PR3-ANCA, kidney and liver parameters, and markers of inflammation, such as erythrocyte sedimentation rate (ESR) and C-reactive protein (CRP), should be performed. Interestingly, in four CRSwNP patients with persistent moderate hypereosinophilia and disease control after the first 12 months of treatment, we adopted a personalized extended dosing interval of 300 mg of dupilumab every three weeks instead of the standard dosing regimen (300 mg of Dupilumab every two weeks). However, dupilumab discontinuation may be avoided to some extent, especially in asymptomatic patients.

Adverse drug reactions to dupilumab in our cohort were generally mild. We observed a case of psoriasis following dupilumab in a patient receiving the drug for CRSwNP, and a second case in a patient with atopic dermatitis. Previous studies reported an increased risk of developing psoriasis in patients treated with dupilumab for atopic dermatitis [46]; psoriatic-like eruptions have also been reported in patients with prurigo nodularis [47] and asthma [48]. The proposed mechanism involved in the development of the psoriatic lesions involves the IL-4-mediated downregulation of Th1 and Th17 [49,50], the increase in IL-23A and IL-17A expression described in psoriatic skin lesions of dupilumab-treated patients with atopic dermatitis [51,52,53], and the evidence that recombinant IL-4 is associated with improved psoriasis [54]. As a consequence, IL-4 signaling blockade may drive cytokine axes toward psoriatic inflammation in some patients.

Another unexpected side effect of dupilumab was the reversal of gray hair (Figure 4) in a male patient who also developed a scalp psoriatic eruption. To our knowledge, this is the first case described in the literature of a patient receiving dupilumab for CRSwNP. Indeed, the sole case of gray hair reversal during dupilumab treatment was reported by Sumitomo et al. [55] in a patient with atopic dermatitis. Interleukin-4 suppresses melanogenesis in normal human melanocytes via the JAK2–STAT6 signaling pathway. Therefore, the dupilumab-induced downregulation of IL-4 may account for this phenomenon [55,56]. Other hair alterations described during dupilumab included worsening and improvement of alopecia [57].

This study is subject to some limitations. The population analyzed is relatively small, and the heterogeneous patient population treated for the various dupilumab indications did not allow subgroup analysis to determine whether the increase in AEC is more common in one disease than another or regarding a potential association between hypereosinophilia and loading-dose regimens. For this reason, future multicenter studies conducted on broader populations treated with dupilumab for different indications are needed to clarify whether risk factors exist for the development of hypereosinophilia, what its long-term impact is, and its relevance in clinical practice.

## 4. Materials and Methods

### 4.1. Study Design and Patients

This was a single-center, real-life, prospective observational study. We evaluated 66 patients with severe asthma and/or CRSwNP and atopic dermatitis undergoing dupilumab treatment followed at the Department of Internal Medicine and Clinical Complexity and Department of Neuroscience, Reproductive Sciences, and Dentistry of the University of Naples “Federico II”. Inclusion criteria were an age ≥ 18 years; asthma and/or severe CRSwNP and/or atopic dermatitis with an indication for biologics treatment according to Global Initiative for Asthma (GINA) [58], the European Academy of Allergy and Clinical Immunology (EAACI) Biologicals Guidelines [40,59] and the European Position paper on rhinosinusitis and nasal polyps (EPOS) 2020 guidelines [60]; dupilumab therapy as their primary biological add-on treatment; available data on sex, date of birth, age of disease onset and diagnosis; and signature of the written informed consent. Exclusion criteria were an age < 18 years, presence of known causes of reactive eosinophilia; previous diagnosis of HES [23] or EGPA [61]; recent (≤4 weeks) oral corticosteroids use before the assessment of AEC; pregnancy and breastfeeding.

We collected both clinical and laboratory data. Clinical items in the analysis were age, gender, presence/absence of type 2 comorbidities, and treatment administered.

This study was conducted in accordance with the Declaration of Helsinki and was approved by the Institutional Review Board of “Federico II” University Hospital (Prot. 75/21, date of approval: 6 May 2021). Before participating, patients signed an informed consent form.

### 4.2. Clinical Scores

ACT, SNOT-22, Smell-VAS, and EASI were assessed according to the type of diagnosis at baseline (T0) and at 6 (T6), 12 (T12), 18 (T18), and 24 (T24) months post-dupilumab initiation. The ACT is a five-item questionnaire that evaluates asthma control over the four weeks prior to the test [62,63]. SNOT-22 is a self-administered questionnaire with 22 items that evaluates symptoms and their impact on QoL in patients with CRSwNP [64]. Smell-VAS was used as a diagnostic tool for the evaluation of olfactory dysfunction [65,66,67,68]. According to EPOS guidelines [60], VAS score may range from 0 to 10, with 0 indicating a normal sense of smell and 10 total anosmia. In addition, patients can be further classified as normosmic-mild (VAS 0–3), moderate (VAS 4–6), and severe olfactory loss (VAS 7–10) [60]. EASI is a tool used to measure the extent and severity of atopic dermatitis, evaluating erythema, papulation, excoriation, and lichenification. It may range from 0 to 72 [69].

### 4.3. Laboratory Assessment

At baseline, the patients underwent allergen SPTs. The SPTs were performed in accordance with the EAACI guidelines using a standard allergen panel (Roxall Italia SRL; Rome, Italy; Lofarma SpA, Milan, Italy). The panel included the following extracts: seven pollens (Gramineae grass pollen (Gramineae mix/Phleum Pratense/Cynodon Dactylon), mugwort, wall pellitory (Parietaria Judaica/Parietaria Officinalis), olea, cypress, birch, and hazel), dander from two animals (cat and dog), two house dust mites (*Dermatophagoides pteronyssinus* and *Dermatophagoides farinae*), tree molds (Alternaria, Aspergillus, and Cladosporium), a negative control (glycerinated saline), and a positive control (histamine). A skin test response was regarded as positive if the wheal diameter was 3 mm greater than that of the glycerinated saline control. Total IgE and specific IgE assay (ImmunoCAP 250; Phadia, Uppsala, Sweden) were also performed. IgE levels were considered positive at a level ≥ 0.35 kU/L.

### 4.4. Blood Eosinophil Count

All blood samples for full blood count and AEC were taken at certified analysis laboratories after at least 4 weeks from the last dose of oral corticosteroids. Complete blood counts were collected monthly during the initial 3 months of dupilumab therapy; however, only AEC at baseline and at 6, 12, 18, and 24 months post-dupilumab initiation were included in the statistical analysis. Eosinophilia was defined as an AEC ≥ 0.5 × 10^9^/L and hypereosinophilia as ≥1.5 × 10^9^/L [21,23]. Patients were further classified according to the severity of the eosinophilia as mild (AEC ≥ 0.5–1.49 × 10^9^/L), moderate (AEC 1.5–5 × 10^9^/L), or severe (AEC > 5 × 10^9^/L) [21,70].

### 4.5. Treatments

After the diagnostic assessment, each patient received personalized recommendations for asthma control and sinonasal symptom management according to the GINA [58] and EPOS 2020 [60] guidelines, respectively. Patients were instructed on the correct use of the devices and behavioral standards to reduce modifiable risk factors. Dupilumab was prescribed to patients with severe bronchial asthma according to the GINA guidelines at the initial dose of 600 mg for the first administration and then 300 mg every other week subcutaneously [58]. Dupilumab was also prescribed for CRSwNP at the dose of 300 mg every other week subcutaneously according to the EPOS 2020 guidelines [60] and American College of Allergy Asthma and Immunology/American Academy of Allergy, Asthma & Immunology (ACAAI/AAAAI) The Joint Task Force on Practice Parameters GRADE guidelines for the medical management of CRSwNP [71]. Treatment outcomes were evaluated at follow-up visits (T0, T6, T12, T18) using clinical (i.e., ACT, SNOT-22, TENPS, Smell-VAS, and EASI) and laboratory (AEC) parameters. In patients with atopic dermatitis, dupilumab was prescribed according to the EAACI guidelines [40] at a loading dose of 600 mg followed by a maintenance dose of 300 mg every 2 weeks.

Adverse events associated with dupilumab were monitored throughout the study to assess the drug’s safety and effectiveness in our cohort and to identify any clinically relevant symptoms. In particular, the patients were evaluated for the following potential adverse events at every follow-up visit: injection site reaction (e.g., erythema, itching, pain, edema, swelling), arthralgia, oral herpes, fatigue, headache, upper airway infections, conjunctivitis, ulcerative keratitis, psoriasis, hair pigmentation, and anaphylaxis.

### 4.6. Data Analysis

Data were summarized by descriptive analysis. Means and standard deviation of the mean (SD) were calculated for continuous variables, while absolute values and frequency (percentage) were calculated for categorical variables.

Values were presented as frequency (number and percentage) and mean ± SD. The level of significance was set at α = 0.05.

Assumption of normality was performed with the Shapiro–Wilk test. Assumption of homoschedasticity was performed with the Levene test. If normality was not rejected at the 0.05 significance level, we used parametric tests.

Analysis of ACT, SNOTT 22, Smell-VAS, EASI and eosinophil levels was performed with independent one-way ANOVA with T0, T6, T12, T18, and T24 visits as a within-subject factor. Post hoc comparisons were performed with independent *t*-tests corrected with the Tukey procedure.

Analysis of dependent variables AEC and total IgE at baseline was performed with an unpaired t-test comparing the two groups: patients developing AEC ≥ 1.5 × 10^9^/L vs. patients with AEC < 1.5 × 10^9^/L.

All analyses were performed with Jamovi, Version 2.3.28. A *p* value less than 0.05 was considered statistically significant for all tests.

## 5. Conclusions

In our population of patients affected by severe T2-related disorders (CRSwNP, severe asthma, and atopic dermatitis), dupilumab administration was entirely effective. Dupilumab-related eosinophilia was common, generally mild, lasting more than 6 months, and spontaneously resolving within 18 months of treatment, whereas moderate, transient, or persistent hypereosinophilia was present in a small group of patients in the absence of symptoms or signs of eosinophilic damage. Prudent monitoring avoided treatment discontinuation while maintaining efficacy and safety. Future research should better focus on the long-term impact of sustained dupilumab-induced eosinophilia and its relevance in clinical practice.

## Figures and Tables

**Table 1:** Baseline characteristics of the study population (N = 66). Absolute blood eosinophil count (AEC), Body Mass Index (BMI), chronic rhinosinusitis with nasal polyps (CRSwNP). ^†^ Positive specific IgE and/or skin prick tests (SPTs) for aeroallergens.

Patients’ Features	Number of Patients (%)
Female gender (n, %)	33 (50%)
Male gender (n, %)	33 (50%)
Caucasian ethnicity (n, %)	65 (98.4%)
Asian ethnicity (n, %)	1 (1.5%)
Age, years (mean ± SD; range)	52.9 ± 17.4; 18–84
BMI (kg/m^2^)	24.8 ± 4.1
Asthma: yes, n (%)	42 (63.6)
CRSwNP: yes, n (%)	54 (81.8%)
Atopic dermatitis: yes, n (%)	6 (9%)
Allergic rhinitis: yes, n (%)	36 (54.5%)
Food allergy: yes, n (%)	9 (13.6)
Total IgE (IU/mL)	313 ± 484
Sensitization to aeroallergens ^†^	17 (25.7%)
Baseline AEC (10^9^/L), mean (SD)	0.507 ± 0.331

**Table 2:** Descriptive analysis of absolute blood eosinophil count (AEC, 10^9^/L) at baseline and 6, 12, 18, and 24 months post-dupilumab initiation in patients presenting with hypereosinophilia (N = 10).

	AEC T0	AEC T6	AEC T12	AEC T18	AEC T24
Mean	0.53	1.74	1.14	1.23	0.90
Standard deviation	0.45	0.10	0.52	0.56	0.61
Range	0.78–1.38	0.57–4.03	0.30–1.80	0.20–2.09	0.98–2.08

**Table 3:** Frequency of adverse events to dupilumab in our cohort (N = 66).

Adverse Events	Number (%)
None	59 (89.39)
Injection site reaction	0 (0)
Conjunctivitis	1 (1.52)
Arthralgia	1 (1.52)
Fatigue	2 (3.03)
Oral herpes	0 (0)
Ulcerative keratitis	0 (0)
Psoriasis	2 (3.03)
Hair pigmentation	1 (1.52)
Anaphylaxis	0 (0)

## Data Availability

The data presented in this study are available on request from the corresponding author.
